# Supplementary material for: New Calamitic Mesogens Exhibiting Aggregation-Induced Emission (AIE)
Source: Materials (Basel). 2024 Jul 20;17(14):3587. doi: 10.3390/ma17143587 (PMC11278991; doi:10.3390/ma17143587)
Supplement: Supplementary file 1 [file materials-17-03587-s001.zip › materials-2554900-supplementary.pdf]

Supplementary Information

New calamitic mesogens exhibiting Aggregation-induced emission (AIE)

Saurav Paul, [a] Bimal Bhusan Chakraborty [a] and Sudip Choudhury * [a,b]

[a] Department of Chemistry, Assam University, Silchar 788011, India.
[b] Centre for Soft Matter, Department of Chemistry, Assam University, Silchar 788011, India.

* Corresponding: sudip.choudhury@aus.ac.in, sudipch1@gmail.com

Contents

1. Synthesis
2. Spectroscopic analysis
3. ^{1}HNMR and ^{13}CNMR Spectra of the synthesised compounds
4. Differential Scanning Calorimetry (DSC) Thermogram of synthesized compounds
5. Electron map of HOMO, LUMO for the Compound 4F3NA12

1. Synthesis

1.1 Synthesis of alkoxy benzoic acid (1)

Ethyl 4-hydroxybenzoate (16.6g, 0.1mol) dissolved in 125ml of 95% ethanol was refluxed for 15 minutes with 10g potassium hydroxide in a round bottomed flask. Then 1-bromoalkane (0.12mol) was added to the refluxing solution followed by potassium iodide in catalytic amount. The system was continued to reflux for 36 hours. Then the excess alcohol was distilled off and 8g of sodium hydroxide dissolved in 200ml water was added to it. Reflux was continued for 10 hours. The reaction mixture was then taken in a beaker and acidified with concentrated hydrochloric acid. The 4-n-alkoxybenzoic acid obtained as a white precipitate was filtered, and washed repeatedly with water and then alcohol (to remove acid, salt and excess alkyl bromide). The crude product was recrystallized from ethanol.

1.2 Synthesis of 4-formyl-3-hydroxyphenyl 4-alkoxybenzoate (2)

The esterification reaction was performed by dissolving two side wings, 2,4-Dihydroxybenzaldehyde (1g, 8.188 mmol) and 4-(dodecyl) benzoic acid (2.51g, 8.188 mmol) in dry dichloromethane (DCM) along with catalytic amount of N, N-dimethyl aminopyridine (DMAP). The mixture was stirred at room temperature for 15 minutes. After that dicylcohexylcarbadimide (DCC) (9.00 mmol) was added to the reaction mixture and stirred for 48 hours. The white precipitate of dicyclohexylurea formed in the reaction mixture was filtered off. Evaporation of the solvent gave the crude product which was then purified by column chromatography using silica gel (60-120 mesh) with hexane: ethyl acetate (9:1) as eluent.

1.3 Synthesis of Schiff-base (4F3NAn, n=12,14,16)

Compound 2 (20 mmol) was dissolved in absolute (abs.) ethanol and heated to reflux along with few drops of glacial acetic acid. To this solution, ethanolic solution of 4-fluoro-3-nitroaniline (20 mmol) was added drop wise. The mixture was then refluxed for 4 hours. The yellow precipitate was separated by filtration and washed several times with ethanol to get the pure product.

2. Spectroscopic analysis

Compound 4F3NAC12

IR (KBr, γmax, cm^{-1}): 2915, 2846, 1735, 1600.

^{1}HNMR ($CDCl_3$, 400MHz): 12.81(s,1H), 8.67(s, 1H), 8.166(d, 2H,J=8.8Hz)), 8.01-7.99(m,1H), 7.60-7.36(m, 3H), 7.01-6.89(m,4H), 4.08(t, 2H, J=6.4Hz), 1.86-0.89(m, 23H).

^{13}CNMR (CDCl3, 100MHz): 164.45, 164.20, 162.29, 156.19, 134.26, 132.80, 129.22, 121.33, 119.93, 118.19, 118.16, 114.79, 114.14, 111.25, 68.79, 32.32, 30.06, 30.05, 30.00, 29.96, 29.76, 26.38, 23.10, 14.54.

Elemental Analysis: calculated (%) for $C_{32}H_{37}FN_2O_6$ (564.26): C, 68.07; H, 6.61; found C, 67.94; H, 6.58

Compound 4F3NAC14

^{1}HNMR ($CDCl_3$, 400MHz): 12.81(s, 1H), 8.67(s, 1H), 8.16(d, 2H, J=8.8Hz), 8.01-7.99(m, 1H), 7.57-7.36 (m, 3H), 7.01-6.88(m, 4H), 4.08(t, 2H, J=6.4Hz), 1.86-0.88(m, 27H).

^{13}CNMR (CDCl3, 100MHz): 164.62, 164.46, 164.21, 162.92, 156.17, 153.05, 152.97, 145.40,145.36, 134.26, 132.80, 129.22, 129.14, 121.32, 119.93, 119.71, 118.20, 118.18, 116.88, 114.79, 114.13, 111.23, 68.79, 32.33, 30.08, 30.06, 30.00, 29.96, 29,77, 29.48, 26.38, 23.10, 14.54

Elemental Analysis: calculated (%) for $C_{34}H_{41}FN_2O_6$ (592.29): C, 68.90; H, 6.97; found C, 68.82; H, 6.88

Compound 4F3NAC16

^{1}HNMR ($CDCl_3$, 400MHz):12.8(s,1H), 8.67(s, 1H), 8.16(d, 2H, J=8.8Hz), 8.01-7.99(m, 1H), 7.60-7.28(m, 3H), 7.01-6.89(m, 4H), 4.08(d, 2H, J=6.4Hz), 1.86-0.88(m, 31H)

^{13}CNMR (CDCl3, 100MHz):164.62, 164.46, 162.93, 156.19, 145.05, 134.26, 132.81, 129.22, 121.33, 119.94, 119.72, 118.19, 118.16, 116.88, 114.79, 114.14, 111.26, 68.79, 32.33, 30.10, 30.06, 29.99, 29.96, 29.77, 29.48, 29.38, 23.10, 14.53.

Elemental Analysis: calculated (%) for $C_{36}H_{45}FN_2O_6$ (620.33): C, 69.66; H, 7.31; found C, 69.82; H, 7.29

3. ^{1}HNMR and ^{13}CNMR Spectra of the synthesised compounds

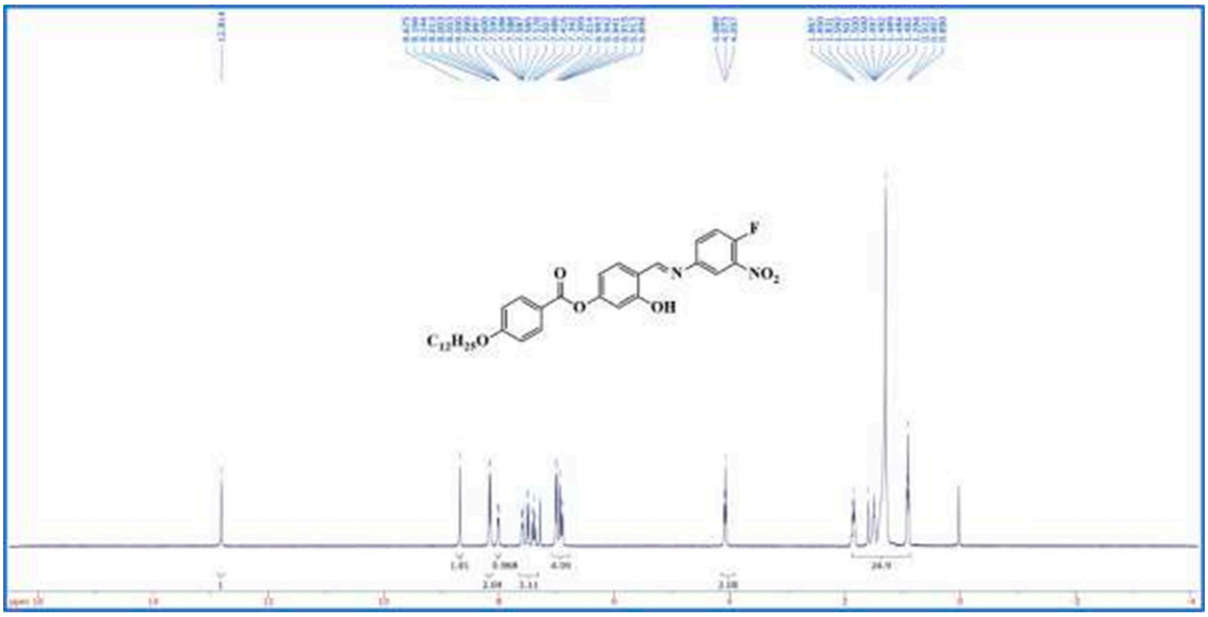

Figure S1: ^{1}HNMR ($CDCl_3$, 400MHz) spectrum of 4F3NA12

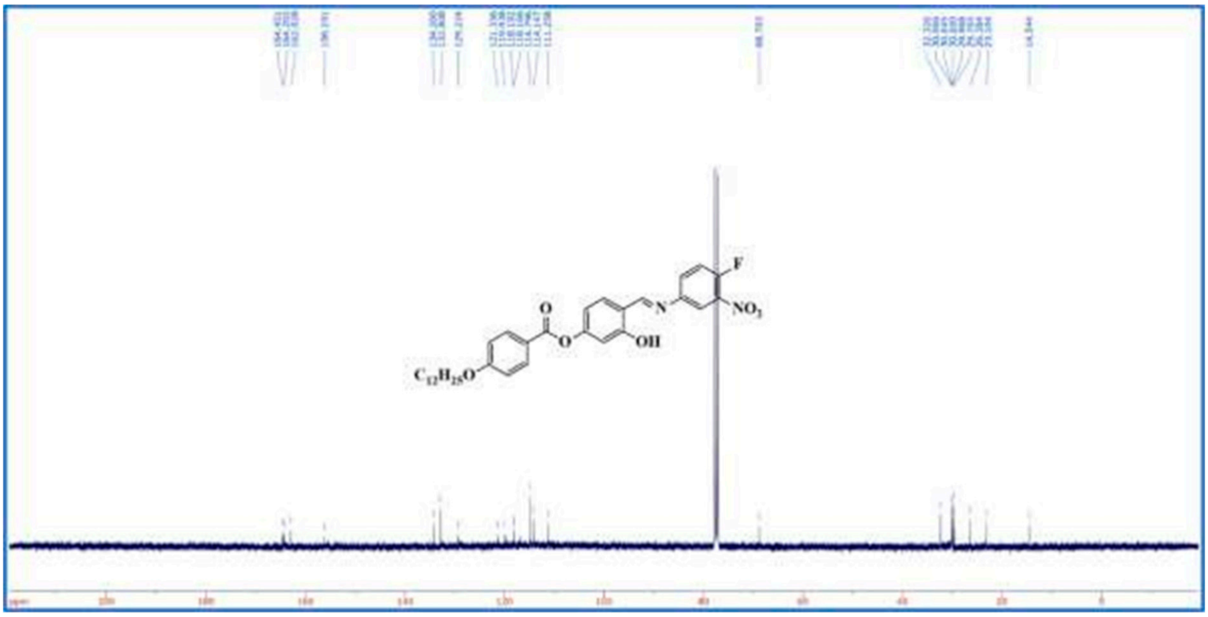

Figure S2: ^{13}CNMR (CDCl3, 100MHz) spectrum of 4F3NA12

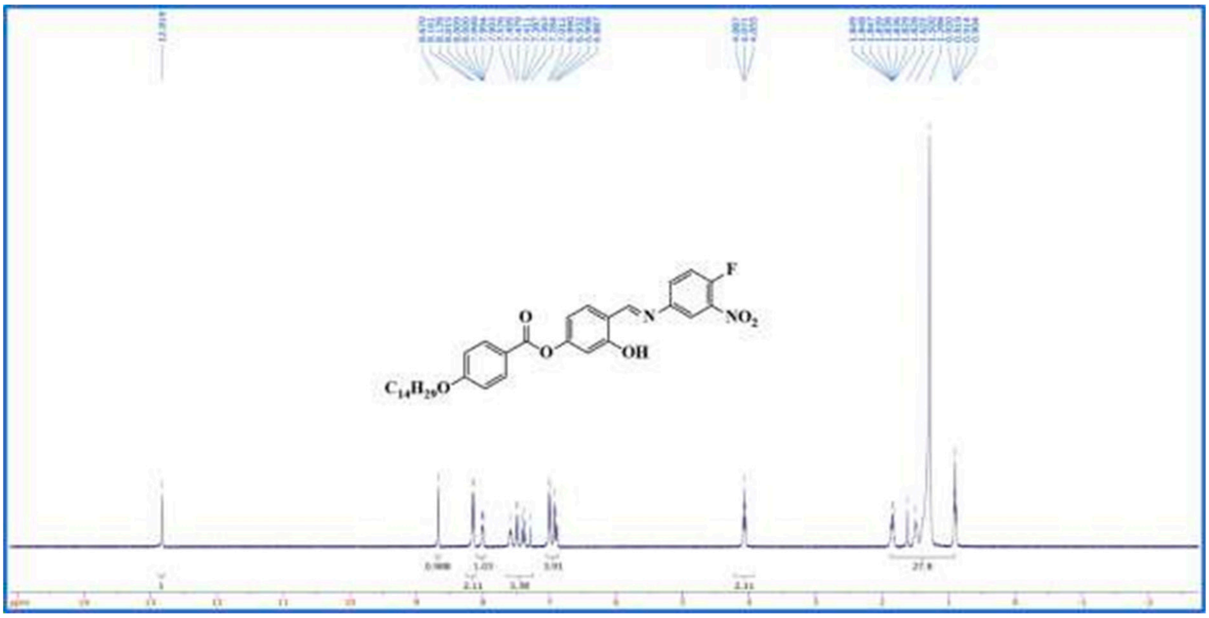

Figure S3: ^{1}HNMR ($CDCl_3$, 400MHz) spectrum of 4F3NA14

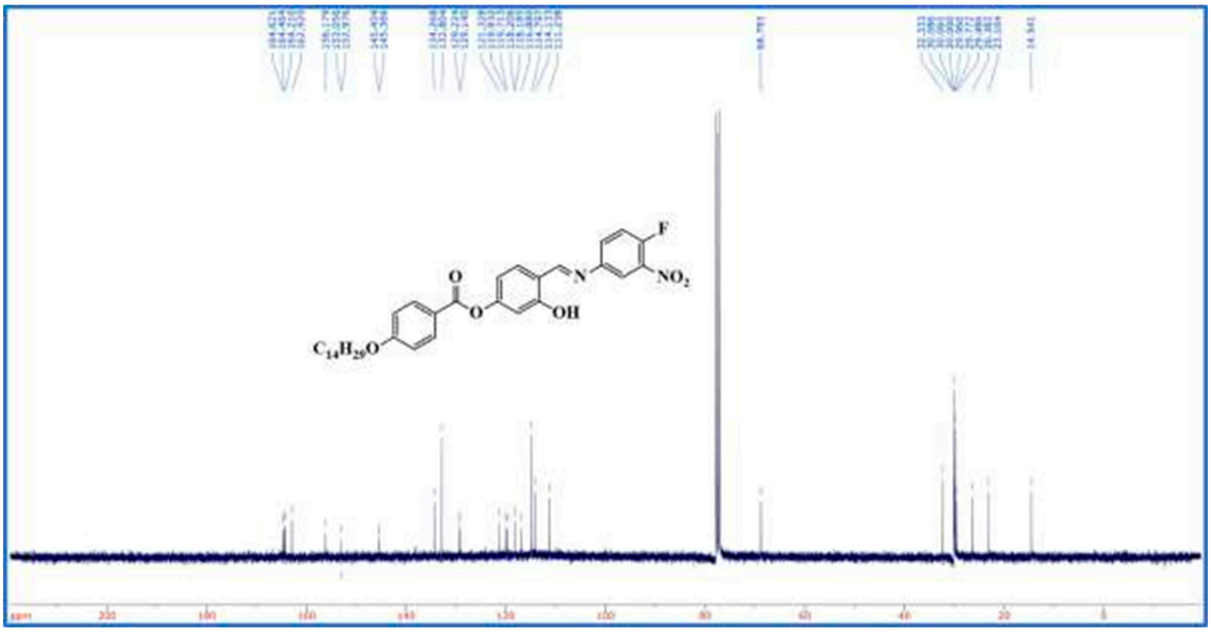

Figure S4: ^{13}CNMR (CDCl3, 100MHz) spectrum of 4F3NA14

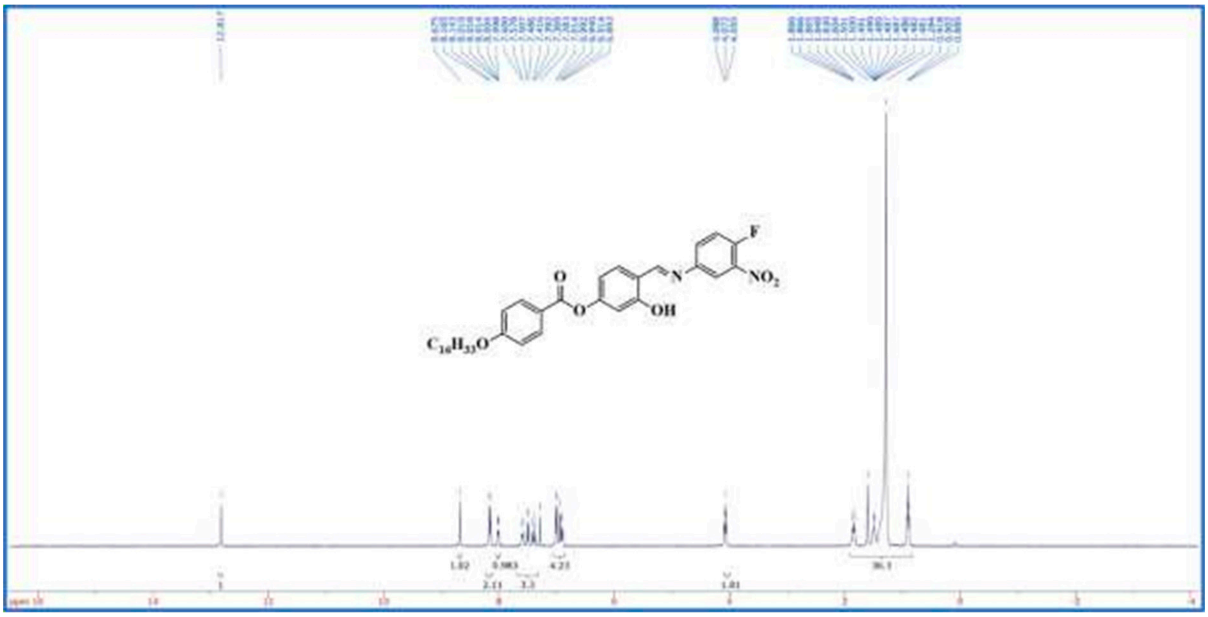

Figure S5: ^{1}HNMR ($CDCl_3$, 400MHz) spectrum of 4F3NA16

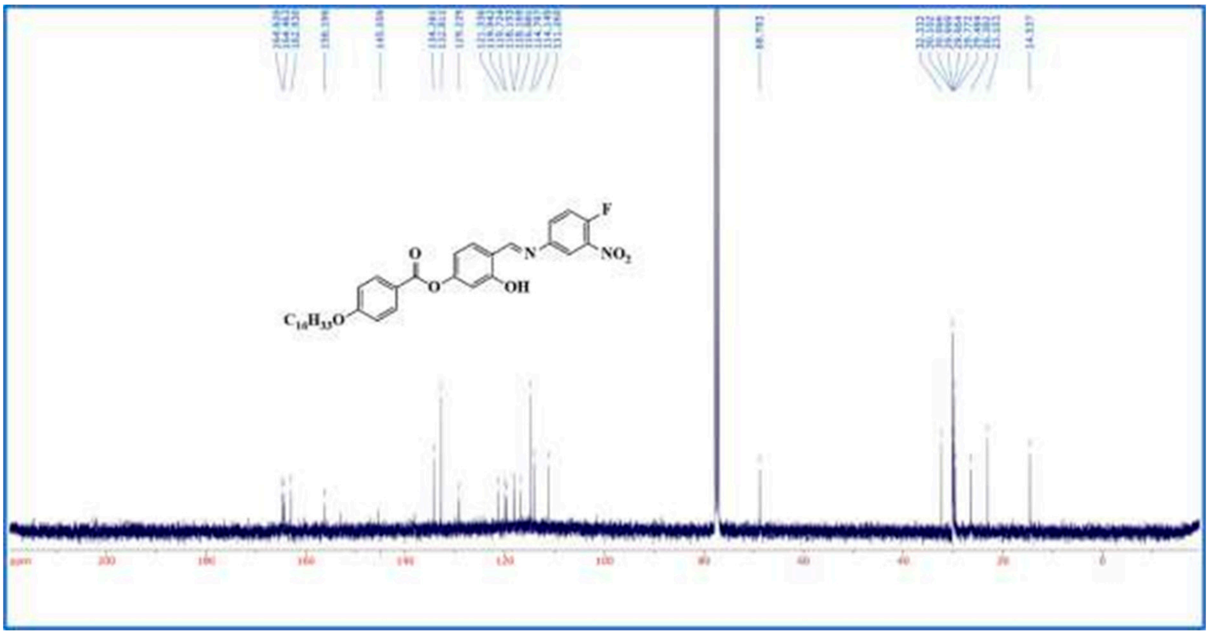

Figure S6: ^{13}CNMR (CDCl3, 100MHz) spectrum of 4F3NA16

4. Differential Scanning Calorimetry (DSC) Thermogram of synthesized compounds

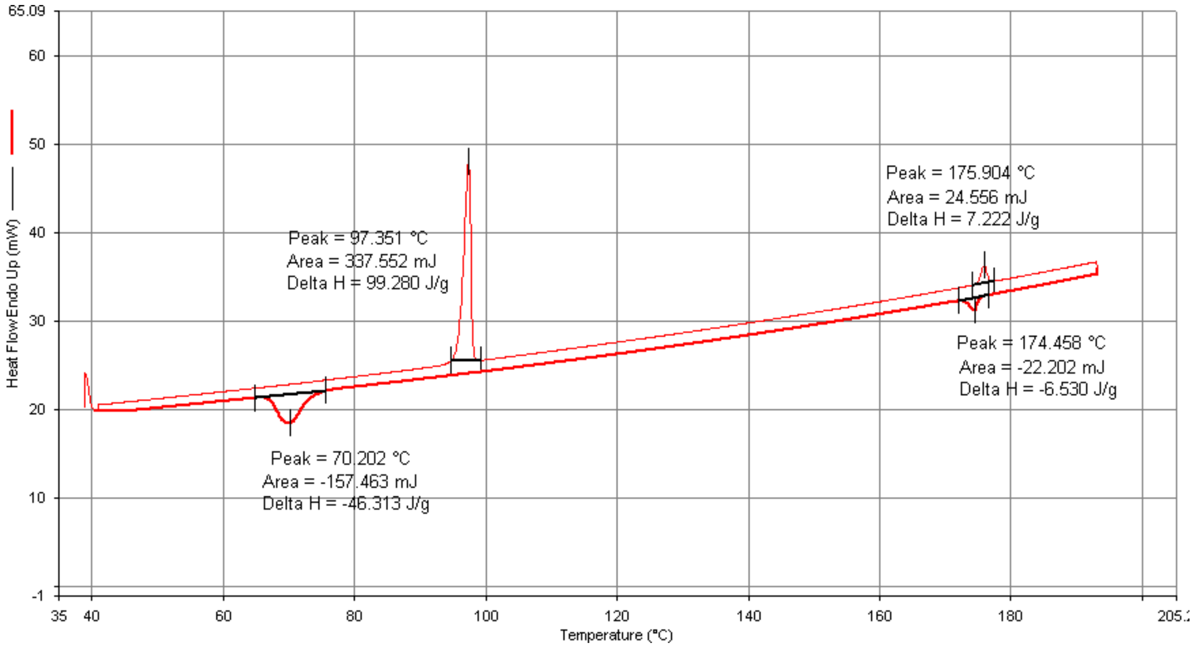

Figure S7: DSC Thermogram of Compound 4F3NA12

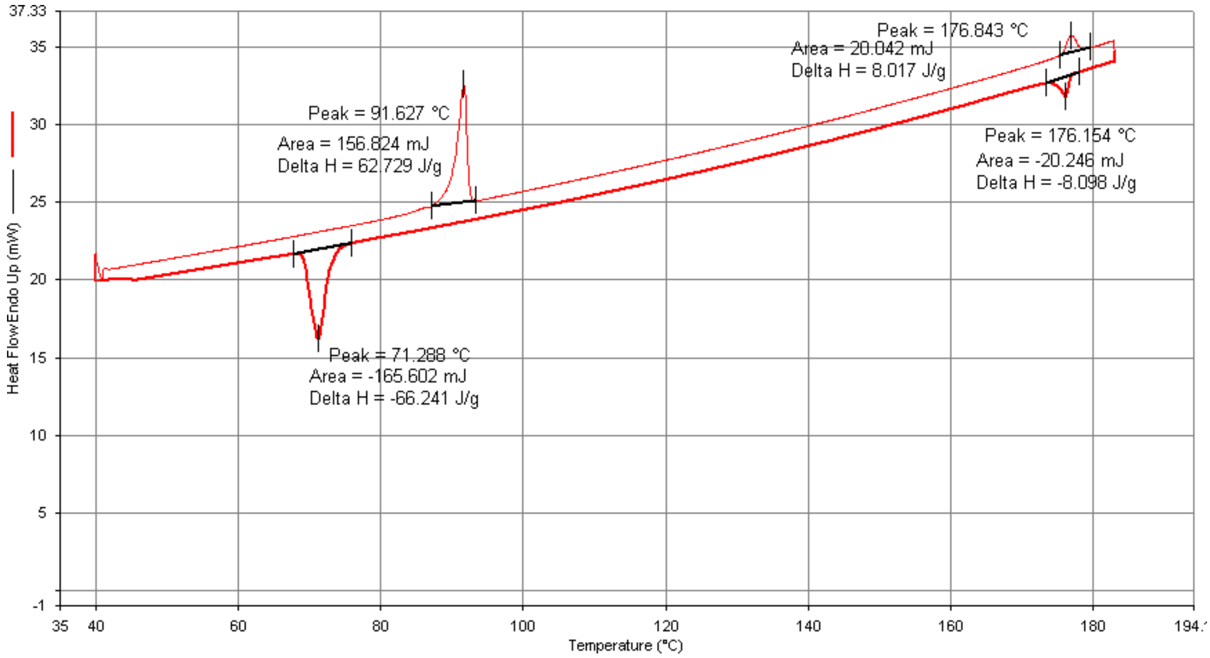

Figure S8: DSC Thermogram of Compound 4F3NA12

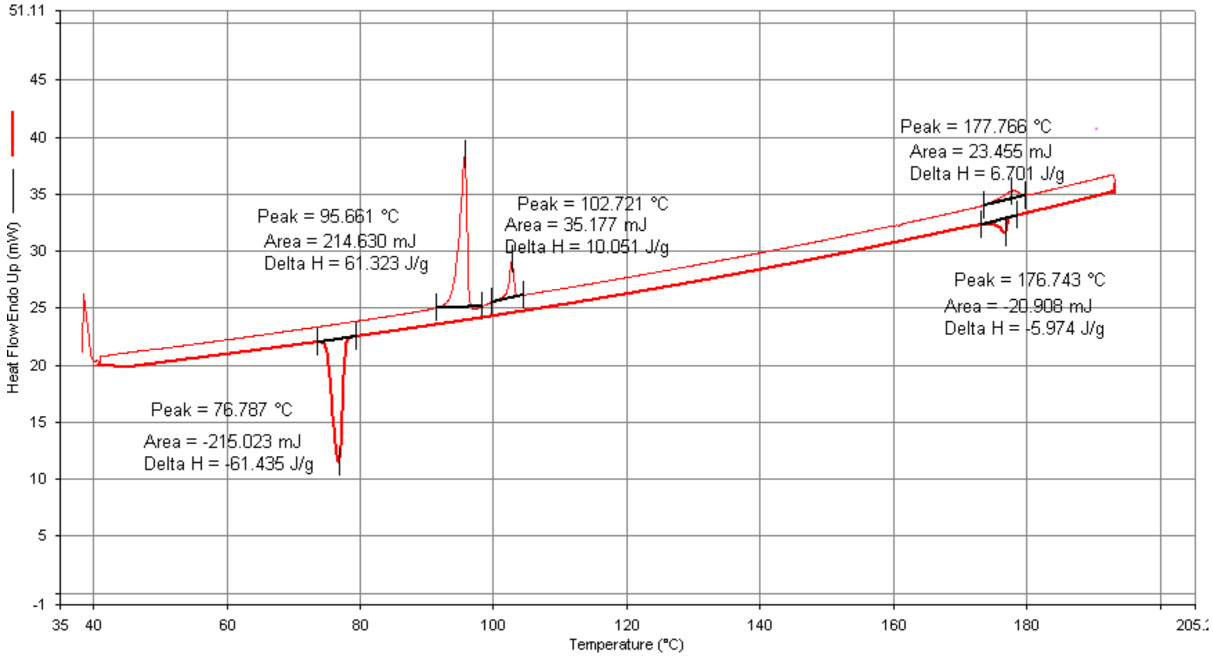

Figure S9: DSC Thermogram of Compound 4F3NA12

5. Computational Study (Compound 4F3NA12)

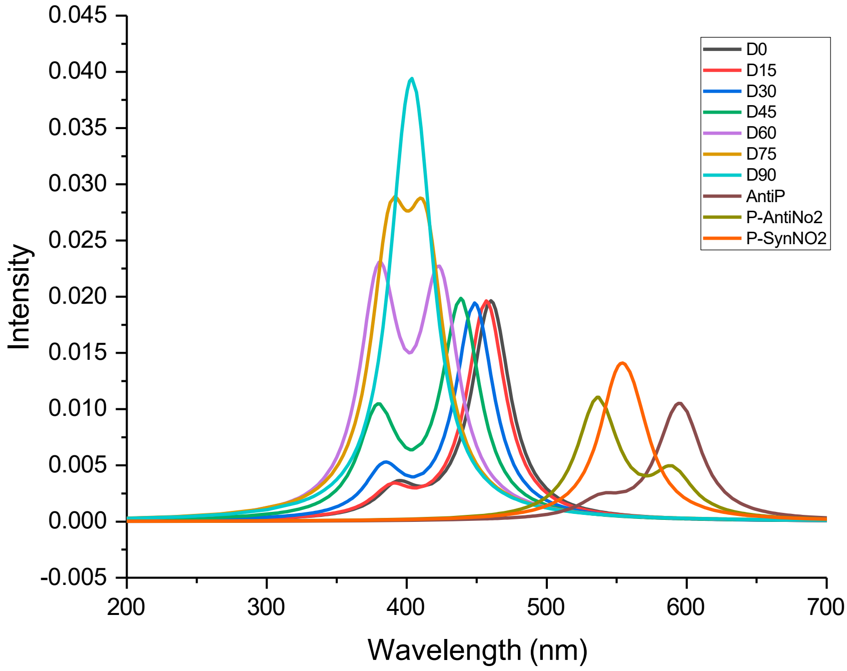

Figure S10: Consolidated plot of all calculated UV-Vis spectra of 4F3NA12

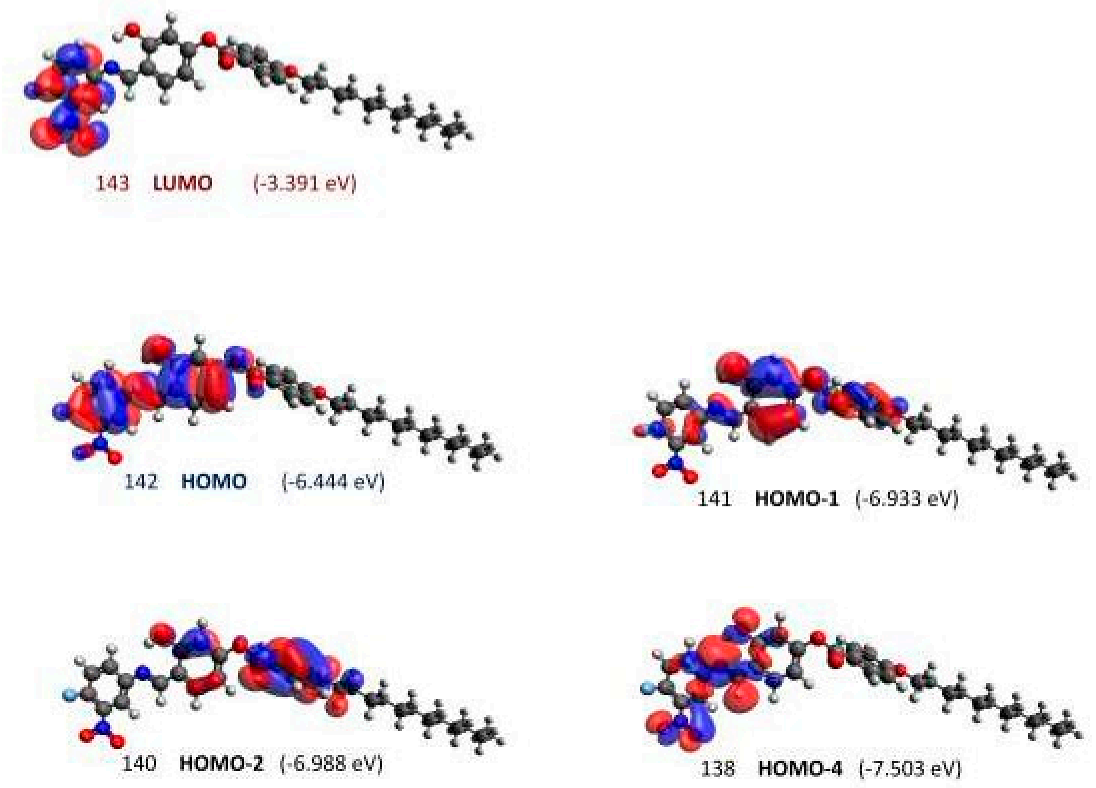

Figure S11: Electron map of MOs from structure D0

P-SynNO2

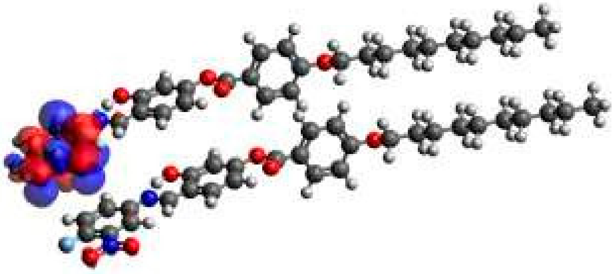

285 LUMO (-3.476 eV)

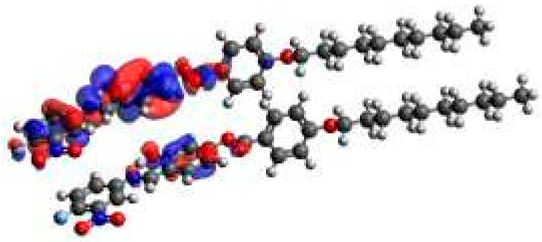

284 HOMO (-6.055 eV)

P-AntiNO2

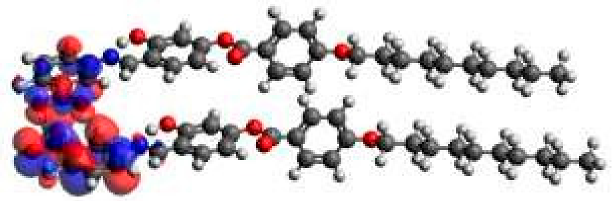

285 LUMO (-3.396 eV)

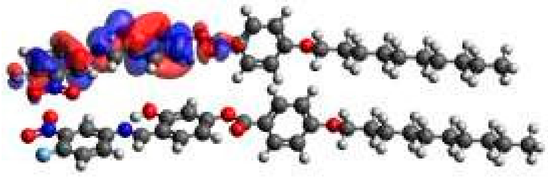

284 HOMO (-5.919 eV)

AntiParallel

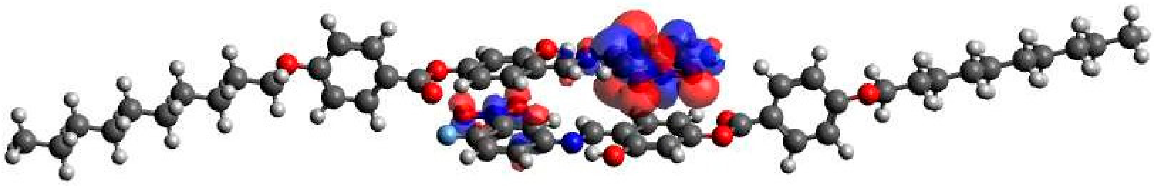

285 LUMO (-3.399 eV)

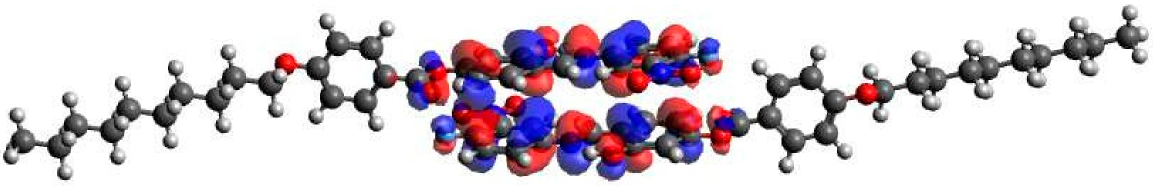

284 HOMO (-5.943 eV)

Figure S12: Electron map of HOMO and LUMO of different orientation of the dimeric system of 4F3NA12

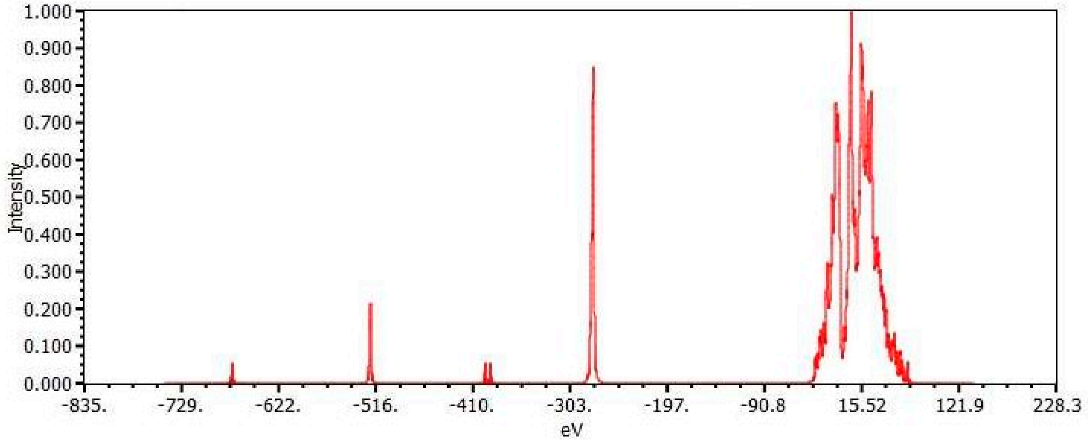

Figure S13: Calculated DOS profile of the Compound 4F3NA12
